# Cardiovascular health assessment in routine cancer follow-up in community settings: survivor risk awareness and perspectives

**DOI:** 10.1186/s12885-024-11912-8

**Published:** 2024-01-31

**Authors:** Kathryn E. Weaver, Emily V. Dressler, Sydney Smith, Chandylen L. Nightingale, Heidi D. Klepin, Simon Craddock Lee, Brian J. Wells, W. Gregory Hundley, Joseph A. DeMari, Sarah N. Price, Randi E. Foraker

**Affiliations:** 1 Department of Social Sciences and Health Policy, Wake Forest University School of Medicine, 1 Medical Center Blvd, Winston-Salem, NC 27157 USA; 2 Department of Biostatistics and Data Science, Wake Forest University School of Medicine, 1 Medical Center Blvd, Winston-Salem, NC 27157 USA; 3 Section on Hematology-Oncology, Wake Forest University School of Medicine, 1 Medical Center Blvd, Winston-Salem, NC 27157 USA; 4 Department of Population Health, University of Kansas Medical Center, Mail Stop 1008, 3901 Rainbow Blvd, Kansas City, KS 66160 USA; 5 Division of Cardiology, Pauley Heart Center, Virginia Commonwealth University, 417 N 11th St 4th Floor, Richmond, VA 23219 USA; 6 Section on Gynecologic Oncology, Wake Forest University School of Medicine, 1 Medical Center Blvd, Winston-Salem, NC 27157 USA; 7 Department of Medicine, Washington University in St. Louis School of Medicine, 660 S. Euclid Ave., MSC 8066-22-6602, St. Louis, MO 63110 USA

**Keywords:** Cancer survivorship, Electronic Health Records, Lifestyle risk factors, Care delivery model

## Abstract

**Background:**

Guidelines recommend cardiovascular risk assessment and counseling for cancer survivors. For effective implementation, it is critical to understand survivor cardiovascular health (CVH) profiles and perspectives in community settings. We aimed to (1) Assess survivor CVH profiles, (2) compare self-reported and EHR-based categorization of CVH factors, and (3) describe perceptions regarding addressing CVH during oncology encounters.

**Methods:**

This cross-sectional analysis utilized data from an ongoing NCI Community Oncology Research Program trial of an EHR heart health tool for cancer survivors (WF-1804CD). Survivors presenting for routine care after potentially curative treatment recruited from 8 oncology practices completed a pre-visit survey, including American Heart Association Simple 7 CVH factors (classified as ideal, intermediate, or poor). Medical record abstraction ascertained CVD risk factors and cancer characteristics. Likert-type questions assessed desired discussion during oncology care.

**Results:**

Of 502 enrolled survivors (95.6% female; mean time since diagnosis = 4.2 years), most had breast cancer (79.7%). Many survivors had common cardiovascular comorbidities, including high cholesterol (48.3%), hypertension or high BP (47.8%) obesity (33.1%), and diabetes (20.5%); 30.5% of survivors received high cardiotoxicity potential cancer treatment. Less than half had ideal/non-missing levels for physical activity (48.0%), BMI (18.9%), cholesterol (17.9%), blood pressure (14.1%), healthy diet (11.0%), and glucose/ HbA1c (6.0%). While > 50% of survivors had concordant EHR-self-report categorization for smoking, BMI, and blood pressure; cholesterol, glucose, and A1C were unknown by survivors and/or missing in the EHR for most. Most survivors agreed oncology providers should talk about heart health (78.9%).

**Conclusions:**

Tools to promote CVH discussion can fill gaps in CVH knowledge and are likely to be well-received by survivors in community settings.

**Trial registration:**

NCT03935282, Registered 10/01/2020

**Supplementary Information:**

The online version contains supplementary material available at 10.1186/s12885-024-11912-8.

## Background

Cancer survivors have almost twice the risk of fatal heart disease compared to the general population, and deaths related to heart disease exceed deaths from the primary cancer for many common cancer types [1–9]. Over 85% of survivors have one or more cardiovascular (CV) risk factors [10–12], increasing their risk of poor CV and cancer outcomes [13–22]. Contributors to heightened CV risk among cancer survivors include: (1) shared mechanisms of cancer and CV disease, including inflammation, tobacco, and obesity; [13, 23] (2) adverse changes in lifestyle factors during cancer treatment (e.g., weight gain) [24, 25]; and (3) cardiotoxic effects of certain cancer treatments [1, 26]. Consequently, the NCCN Clinical Practice Guidelines in Oncology (NCCN Guidelines) for Survivorship recommend cardiovascular risk assessment and counseling for all cancer survivors throughout the survivorship continuum [27]. ASCO guidelines also recommend CVH assessment and counseling, specifically:

*Clinicians should regularly evaluate and manage cardiovascular risk factors such as smoking, hypertension, diabetes, dyslipidemia, and obesity in patients previously treated with cardiotoxic cancer therapies. A heart-healthy lifestyle, including the role of diet and exercise, should be discussed as part of long-term follow-up care* [28].

To effectively implement these and similar guidelines to prevent CV morbidity in cancer survivors, it is critical to understand survivors’ current understanding of their heart health, as well as their perspectives on addressing CV risk during routine oncology care. In our pilot work with breast cancer survivors treated at an academic medical center, we uncovered gaps in survivors’ knowledge of heart health, as well as their attainment of ideal cardiovascular health (CVH) [29]. More than half of these survivors reported not knowing their level for one or more CVH factors, and less than 50% had ideal blood pressure (BP), body mass index (BMI), cholesterol, diet, and physical activity per American Heart Association guidelines [30]. Considering that the majority of survivors receive their care in community settings [31], it is important to understand whether these results generalize to a more diverse group of cancer patients seen in community oncology practices for routine follow-up.

We present baseline data from an ongoing hybrid effectiveness-implementation study (WF-1804CD) of a novel electronic health record (EHR)-embedded heart health assessment tool, Automated Heart-Health Assessment (AH-HA) [13, 29, 32, 33]. The AH-HA trial collects data on CVH factors and care coordination among cancer survivors receiving routine survivorship care in NCI Community Oncology Research Program (NCORP) outpatient oncology practices [34]. The AH-HA tool assesses the American Heart Association’s Life’s Simple 7 CVH factors [35] (body mass index, smoking status, blood pressure (BP), total cholesterol, hemoglobin A1c (HbA1c) or blood glucose, physical activity, and diet) using a combination of self-report and EHR data. The objectives of these baseline analyses were to: (1) assess the CVH profiles of post-treatment cancer survivors receiving routine follow up care in community oncology settings, (2) compare self-reported and EHR-based categorization of CVH factors, and (3) describe cancer survivors’ perceived health risks, motivation to improve CVH, as well as the perceived appropriateness of addressing CVH during outpatient oncology encounters.

## Methods

### Study sample

This cross-sectional analysis utilized baseline data from participants in an ongoing study to examine the effects of the AH- HA tool among survivors receiving routine follow-up care in community oncology settings, using a group-randomized trial design [34]. This study was conducted in partnership with 9 outpatient oncology practices affiliated with the Wake Forest NCI Community Oncology Research Program (WF NCORP) Research Base (NCT03935282, registered 10/01/2020). Eligible survivors included those presenting for routine cancer-related follow-up care ≥ 6 months post-potentially curative treatment for breast, prostate, colorectal, or endometrial cancers or Hodgkin and non-Hodgkin lymphomas; ongoing hormonal therapies such as tamoxifen, aromatase inhibitors (with or without adjuvant CDK 4/6 inhibitors such as abemaciclib), or androgen deprivation were allowed. To be eligible, survivors had to be free of disease at their last medical visit for all cancers. The trial was available in English and Spanish. Local NCORP site staff contacted potentially eligible survivors prior to their appointment via mail, telephone, e-mail, patient portals, or in-person to provide study information, screen, and ascertain interest in participating.

### Data collection

The study was approved by the NCI Central Institutional Review Board (CIRB), and all participants provided informed consent prior to participation. Patient surveys were generally administered using a web-based platform, with paper and phone as back-up options. Enrolled survivors completed a baseline assessment (~ 15 min) within two weeks prior to their regularly scheduled follow-up clinic visit and then a second survey immediately following their visit. A trained research staff member completed the EHR chart abstraction form following the designated clinic visit. Survivors received a $10 gift card after completing the post-visit survey.

### Measures

Survivor CVH factors were collected via both self-reported numerical values [weight, height, smoking status, blood pressure (BP), total cholesterol, hemoglobin A1c (HbA1c), physical activity, and diet] and the most recent value abstracted from the EHR. Physical activity and diet were not available from the EHR. In the case where survivors were unsure of the numerical value for a heart health factor, they could select “I don’t know” as an option. Self-report items (Supplementary Material 1) were based on prior work in both primary care and oncology [13, 29]. Each factor for both methods (EHR and self-report) was scored as ideal, intermediate, poor, or missing/unknown according to the American Heart Association Simple 7 framework [30]. Self-report and EHR were considered concordant if they resulted in the same categorization (e.g., ideal, intermediate, poor). Survivor knowledge of CVH and perceived importance and appropriateness of heart health discussions during oncology care were evaluated with 6 questions used in prior work [29] assessing: 1) confidence in understanding risk of heart disease, 2 & 3) understand (or plan to take) steps needed to maintain or improve heart health, 4 & 5) perception that cancer (or heart disease) pose a risk to health, 6) importance of talking with oncology provider about heart health, and 7) oncology providers should talk to patients about heart health. These questions were rated on a 5-point Likert scale from strongly agree to strongly disagree. Survivors also reported age, gender, race/ethnicity, years of education, marital status, and visits to primary care and cardiology within the past six months. Key medical information from the EHR, including cancer type, stage, date of diagnosis, and cancer treatment was abstracted by research staff at each site. Cancer treatments with cardiotoxic potential were classified according to Herrmann 2020 [36]; specifically, those cancer therapies classified as having very common (> 10%) frequency of either cardiac or arrhythmia toxicity were defined as high cardiotoxicity potential therapies. We also collected information on treatments with less frequent occurrence of cardiotoxicity. Non-cancer cardiovascular comorbidities included: high cholesterol, hypertension, obesity, diabetes with and without complications, heart failure, and atherosclerotic vascular disease (ASCVD, inclusive of heart disease, myocardial infarction, peripheral vascular disease, and cerebrovascular disease/stroke). Rural residence is defined as residing in a zip-code that qualifies as rural according to the Federal Office of Rural Health Policy definition [37]. 

### Statistical considerations

Descriptive statistics of count (frequency) and mean (standard deviation) were used to characterize baseline patient demographics, healthcare utilization, and cardiovascular comorbidities categorical and continuous variables respectively. Counts and frequencies were also used to characterize cardiovascular health perceptions and receipt of cardiotoxic cancer treatments. We classified the AHA Simple 7 cardiovascular health factors from both self-report and the EHR as ideal, intermediate, or poor according to the American Heart Association Simple 7 rubric [30].

## Results

### Sample characteristics

Data for the present analyses were available from 502 enrolled survivors (10/1/2020-10/21/2022) with complete baseline self-report and medical record data from 8 enrolling practices in 7 states. At the time of analysis, we had screened 590 participants; 2 were ineligible, 77 declined participation, and 9 did not respond. Breast cancer survivors comprised most of the sample (79.7%, *n* = 400), with smaller proportions of endometrial (10.6%, *n* = 53), colorectal (5.8%, *n* = 29), lymphoma (2.4%, *n* = 12), and prostate (0.2%, *n* = 1) cancers (Table 1). The mean time since cancer diagnosis was 4.21 years (SD = 3.32). Diagnosis dates for survivors’ most recent cancer ranged from 10/1995 to 12/2021. Females comprised 95.6% (*n* = 480) of the sample; participants identified primarily as non-Hispanic/Latino, white (86.3%, *n* = 433). A little more than half of survivors were between 40 and 65 years of age (51.2%, *n* = 257), with 4.0% (*n* = 20) under 40 and 44.8% (*n* = 225) 65 years and older.

**Table 1:** Demographic and cancer characteristics of enrolled post-treatment cancer survivors (*N* = 502)

	N	%
**Age**		
18–39	20	4.0
40–64	257	51.2
65–74	175	34.8
75+	50	10.0
**Female Gender**	480	95.6
**Race/ Ethnicity**		
American Indian or Alaska Native	3	0.6
Asian	4	0.8
Black/African American	35	7.0
More than one race	7	1.4
White, non-Hispanic or Latino	433	86.3
White/Other/Unknown, Hispanic or Latino	17	3.4
Other/Unknown, Not Hispanic or Latino	3	0.6
**Rural Residence**		
Non-metro#	89	17.7
**Education**		
High School or Less	107	21.3
Some College (including vocational/ technical)	170	33.9
College degree or more	223	44.4
Prefer not to answer	2	0.4
**Marital Status**		
Married/Living as Married	340	67.7
Single, Divorced, Separated, or Widowed	161	32.1
Prefer not to answer	1	0.2
**Cancer Type**		
Breast	400	79.7
Colorectal	29	5.8
Endometrial	53	10.6
Prostate	1	0.2
Lymphoma	12	2.4
Multiple Cancer Types	7	1.4
**Time Since Diagnosis (years)**		
Mean (SD), Range	455	4.21 (3.32), 0.52–26.72
**AJCC Cancer Stage for Most Recent Cancer**		
0	25	5.0
1	210	41.8
2	117	23.3
3	64	12.8
4	3	0.6
Unknown/NA	83	16.5
Health Care Utilization* (self-reported)		
PCP (last 6 months)	397	80.0
Cardiologist (last 6 months)	71	14.2
**How confident are you filling out medical forms by yourself?**		
Extremely	352	70.1
Quite a bit	103	20.5
Somewhat/A little bit/ Not at all	47	9.4
**During the past 4 weeks, did you have enough money to meet the daily needs of your family?**		
No	17	3.4

#Defined as residing in a zip-code that qualifies as rural according to the Federal Office of Rural Health Policy definition

*Six participants reported not knowing if they had seen a primary care provider in the last six months; Two participants reported not knowing if they had seen a cardiologist in the last six months

### Objective 1: cardiovascular health profiles

#### Comorbidities

The most common cardiovascular comorbidities (Table 2) were high cholesterol (48.3% *n* = 241), hypertension or high BP (47.8%, *n* = 240), obesity (33.1%, *n* = 166), and diabetes (20.5%, *n* = 103).

**Table 2:** Cardiovascular comorbidities and cancer therapy with cardiotoxicity potential among post-treatment cancer survivors (*N* = 502)

Cardiovascular Comorbidities	N	%
High Cholesterol^1^	241	48.3
Hypertension or high BP	240	47.8
Obesity^2^	166	33.1
Diabetes	103	20.5
Atherosclerotic Vascular Disease (ASCVD)^2^**	67	13.4
Heart Failure	15	3.0
Receipt of Cancer Treatment with High Cardiotoxicity Potential***	153	30.5
^1^*n*=499; ^2^*n*=501		

**ASCVD includes Heart Disease, Peripheral Vascular Disease, Myocardial Infarction, and Cerebrovascular Disease

***classified according to Herrmann 2020 [36]

#### Receipt of cancer treatment with cardiotoxic potential

Almost a third of survivors (*n* = 153, 30.5%) received at least one cancer treatment with high cardiotoxicity potential (Table 2). Anthracycines were the most common (*n* = 109, 21.7%), followed by monoclonal antibodies (*n* = 53, 10.6%, Supplementary Material 2). Receipt of cancer treatment with lower risk of cardiotoxicity was common in the sample; 93.8% of survivors received treatment with at least some cardiotoxicity potential (Supplementary Material 2).

#### Cardiovascular factors

**Fig. 1:**
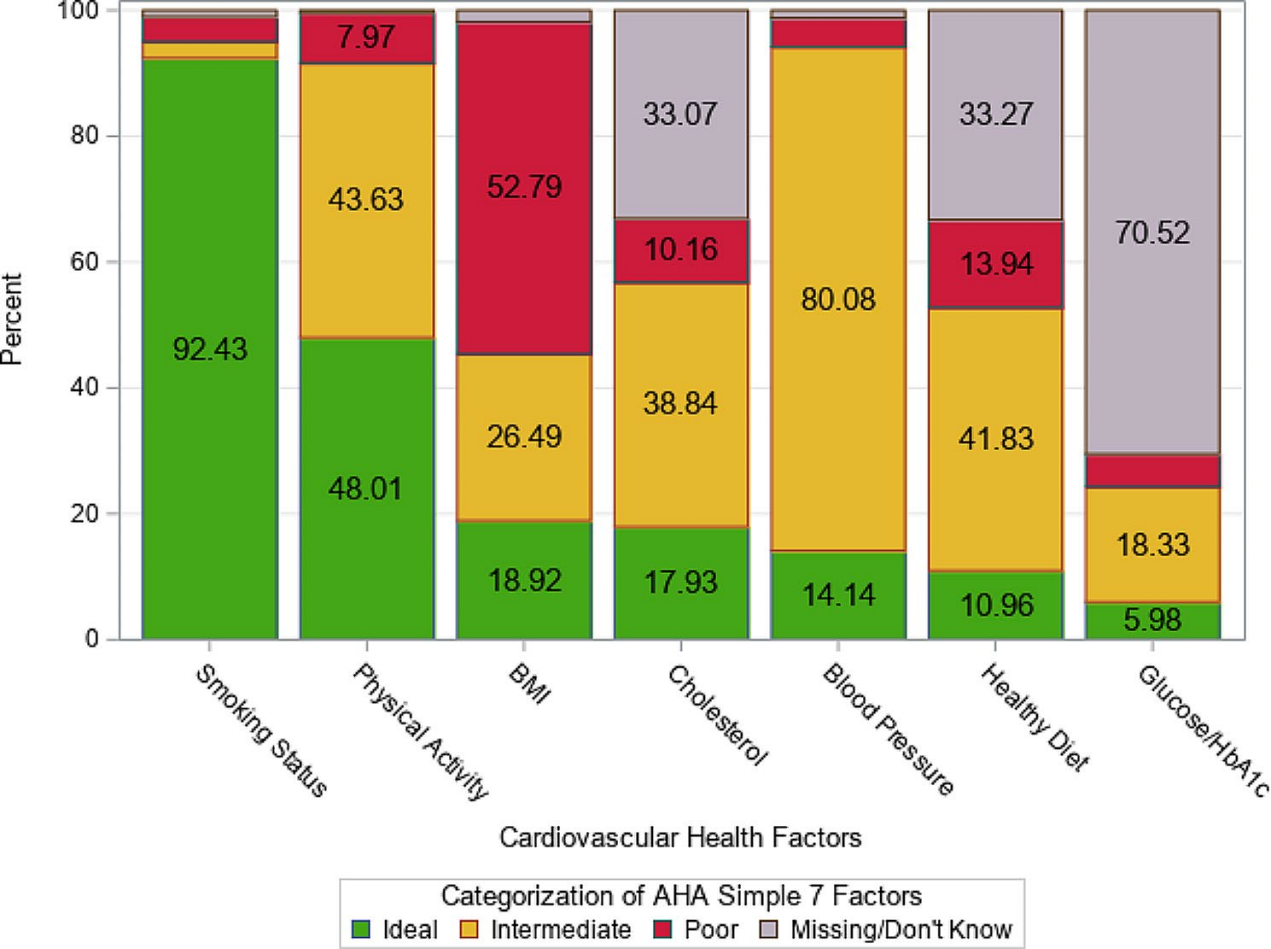
Simple 7 cardiovascular health factors among post-treatment cancer survivors (*N* = 502). American heart association simple 7 physical activity and diet components are self-reported prior to a routine oncology visit; all other components are from the electronic health record. Missing/Don’t know means EHR missing and/or survivor unknown

Using the AHA Simple 7 ratings (ideal, intermediate, poor), most survivors (92.4%, *n* = 464) had an ideal smoking status; less than half of survivors (48%, *n* = 241) reported ideal levels of physical activity (Fig. 1). Smaller proportions of survivors had ideal levels for BMI (18.9%, *n* = 95), cholesterol (17.9%, *n* = 90), BP (14.1%, *n* = 71), and glucose/ HbA1c (6.0%, *n* = 207). More than half (52.8%, *n* = 265) of survivors had poor BMI. Among survivors with available data (i.e., survivor known and/or available from EHR), intermediate was the most common level for cholesterol (38.8%, *n* = 195), BP (80.1%, *n* = 402), a healthy diet (41.8%, *n* = 210), and glucose/ HbA1c (18.3%, *n* = 92).

### Objective 2: CVH concordance between survivor self-report and EHR

**Fig. 2:**
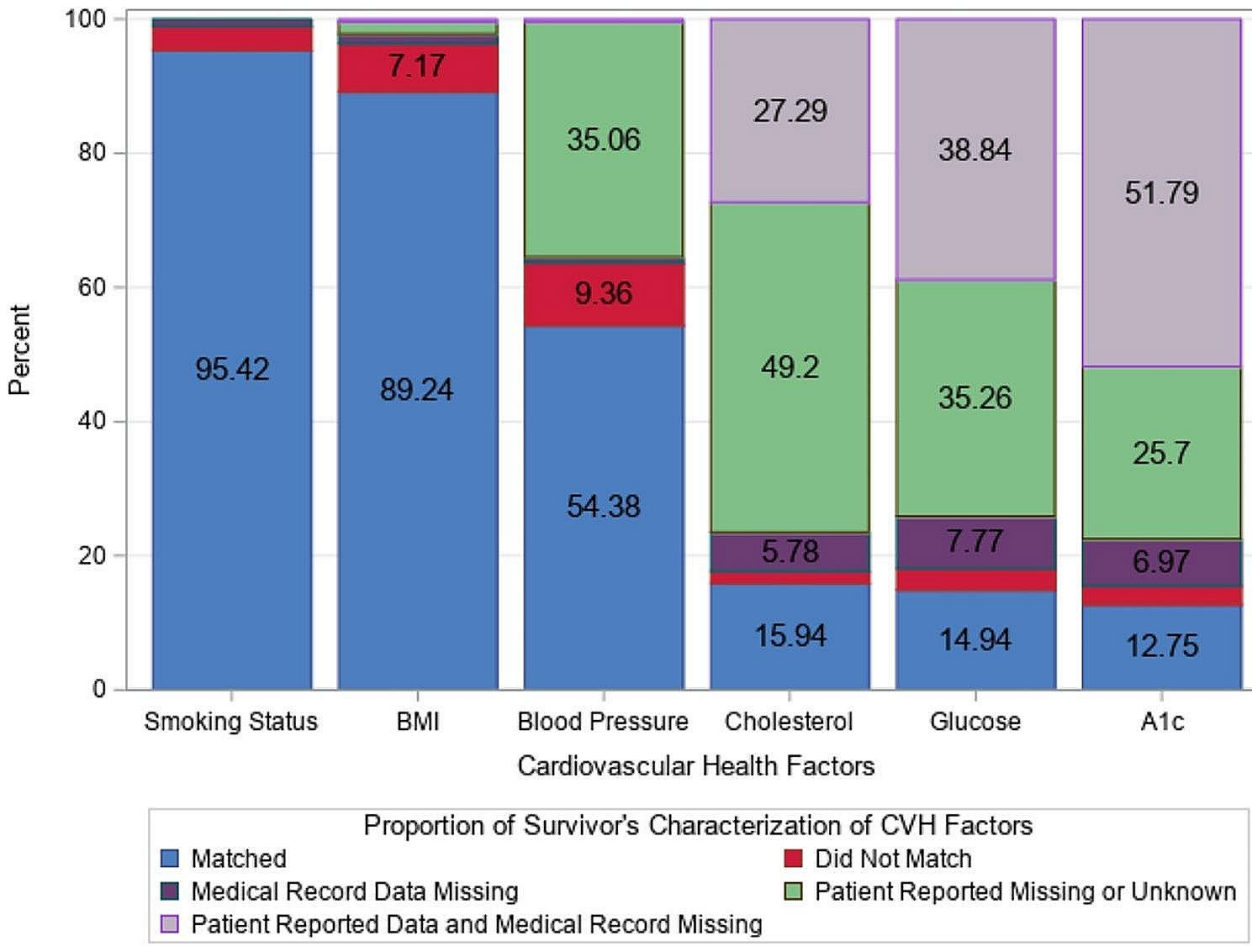
Concordance of survivor cardiovascular factors from self-report and EHR (*N* = 502). American heart association simple 7 cardiovascular health factors were classified according to the 2011 framework [30]. These five components were self-reported prior to a routine post-treatment oncology visit and abstracted from the electronic health record at the same visit. BMI = body mass index, AIC = hemoglobin A1c

Most participants (> 80%) had blood pressure, smoking status, and BMI values reported from the same day as their designated clinic visit (median number of days between appointment day and reported day = 0 for each measure); the median number of days between the designated clinic visit and the test reported day was longer for cholesterol (median = 205 days), glucose (median = 121 days), and HbA1c (median = 173 days), which are less frequently measured. The proportion of survivors with self-report and EHR concordance for categorization varied across CVH factors assessed (Fig. 2). There was concordance for most survivors with smoking status (95.4%, *n* = 479) and BMI (89.2%, *n* = 448); 54.4% (*n* = 273) had concordance for BP. Concordance rates were low for cholesterol (15.9%, *n* = 80), glucose (14.9%, *n* = 75), and HbA1c (12.8%, *n* = 64) primarily because self-reported categorization was unknown and/or values were missing in the EHR. Many survivors reported that they were unaware of their BP (35.1%, *n* = 176), cholesterol (49.2%, *n* = 247), glucose (35.3%, *n* = 177), and HbA1c (25.7%, *n* = 129). Risk category was unknown (survivor reported “don’t know” and missing from EHR) for a substantial number of survivors for cholesterol (27.3%, *n* = 137), glucose (38.8%, *n* = 195), and HbA1c (51.8%, *n* = 260).

### Objective 3: cardiovascular health perceptions among post-treatment cancer survivors

**Fig. 3:**
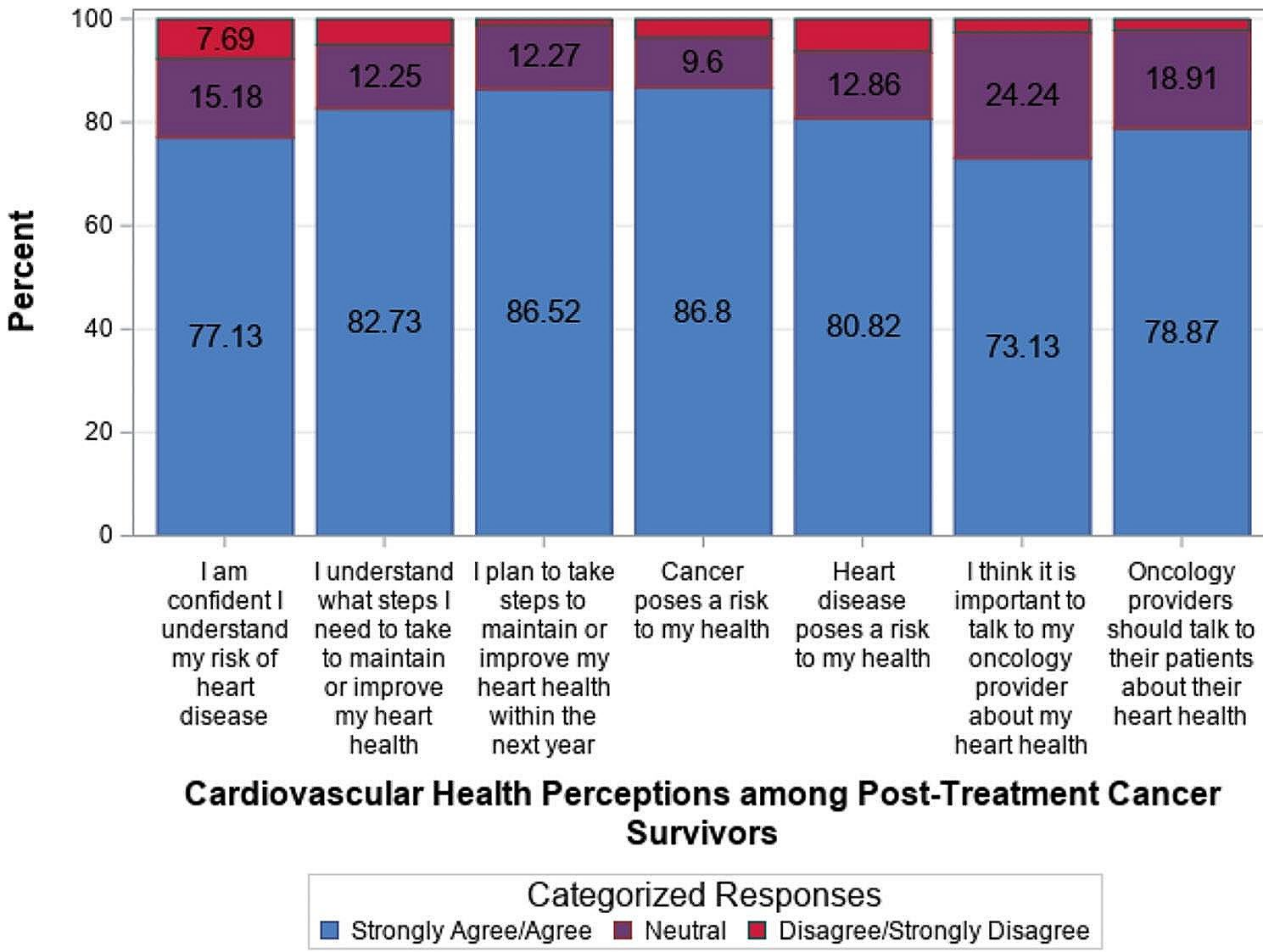
Cardiovascular health perceptions among post-treatment cancer survivors (*N* = 502)

The majority of survivors strongly agreed/ agreed with each of the cardiovascular health perception items ranging from 73.1% (*n* = 362) (*important to talk to my oncology provider about heart health*) to 86.8% (*n* = 434) (*cancer poses a risk to my health*) (Fig. 3). Few survivors (1.2 − 7.7%, *n* = 6–38) strongly disagreed/disagreed with the cardiovascular health perception items.

## Discussion

There was considerable burden from cardiovascular comorbidities and risk factors among the post-treatment cancer survivors treated in community settings enrolled in this study. Nearly one third received at least one cancer treatment with high cardiotoxicity potential, and a large majority received a treatment with at least some cardiotoxicity potential. Although most survivors had an ideal smoking status, many had intermediate or poor levels for the other AHA Simple 7 cardiovascular factors. Many survivors correctly self-reported smoking andBMI status; however, most survivors had BMI values in the intermediate or poor level. Several of the CVH factors (e.g., cholesterol, glucose, HbA1c) were not known among survivors and not reported in the EHR. For the relatively small proportion of survivors (< 10% for each CVH factor) whose EHR values and self-reported characterization did not match, self-reported values were generally better than those documented in the EHR (ranging from 50% for HbA1c to 88.9% for cholesterol). Survivors and clinicians cannot take appropriate action if they are unaware of or underestimate cardiovascular health risk factors. These results mirror other studies which show similar rates of cardiovascular comorbidities [6, 33], and highlight the importance of clinical practice guidelines recommending cardiovascular health assessment as part of routine follow-up care for cancer survivors [27, 28]. We also found that most survivors were motivated to improve their CVH, perceived that there are risks posed by cancer and heart disease, and agreed that oncology providers should talk to patients about their CVH. This suggests survivors will be receptive to efforts to implement CVH assessment and management into their oncology care.

Very few cancer survivors in this sample and others [38] report ideal CVH, suggesting that most survivors routinely seen for follow-up care could benefit from CVH assessment and education, with potential long-term improvements in survival and risk of CV disease [39–42]. An emerging consensus suggests optimal management of a cancer survivor should be done collaboratively using a team approach between oncologists, primary care providers, and cardiologists, among others [43–45]. Survivors want oncologists to address their heart health. While oncologists are aware of the cardiotoxicity profile of cancer treatments, especially those with the highest associated risk, they may not routinely incorporate management of CV risk factors into follow-up care once cancer treatment is completed, assuming this issue will be managed by others such as primary care providers. Nearly 80% of the survivors in our study had a recent primary care visit, suggesting the availability of primary care for primary management of cardiovascular risk. By contrast, only 14% of those in our study received care from a cardiologist in the prior 6 months.

Regardless of whether they were seen by primary care or cardiology, many survivors were unaware of key CVH factors, suggesting an opportunity for education and brief intervention. Innovative tools, such as the one being tested in the current study, can improve the implementation of clinical practice guidelines calling for a CVH assessment in the oncology setting. Work-flow compatible strategies for collecting information from patients about CVH factors not routinely available from the EHR (i.e., physical activity & diet) are needed. Other CVH factors were often missing in the EHR (i.e., cholesterol and glucose/HbA1c), and survivors frequently reported not knowing their values for these same variables. Patient’s CVH cannot be effectively assessed, and their risk factors appropriately managed, without these important data elements. Public health campaigns such as the AHA’s “Know Your Numbers” [46] may be valuable for cancer survivors, as would targeted efforts to fill in missing test data by primary care and oncology providers.

There were numerous strengths to this study, including a large sample of survivors seen in outpatient community oncology practice. We included many cancer types to reflect the variety of providers seen for long-term follow-up care and recruited a sample diverse with respect to age. We also had staff on site at community practices to abstract medical information from the EHR to improve data quality and completeness. Specific limitations of the study are discussed below. During our funding period, the AHA also updated its framework for CVH by adding sleep as the eighth modifiable risk factor to Life’s Simple 7, creating Life’s Essential 8 [47]. Although this addition does not affect the results of the present analysis [48], we plan to prospectively collect sleep data (average hours per night) at later time points, providing information which can inform routine assessment and management of sleep health, an understudied issue in survivorship care [49]. 

Our baseline data demonstrate a high prevalence of multiple cardiovascular comorbidities among cancer survivors, as well as the desire to discuss CVH with their oncologist. The AH-HA platform delivered through the EHR is one promising strategy to facilitate the delivery of guideline concordant cardiovascular health assessment and discussion in outpatient oncology care. Such efforts to combine health information technology tools and cancer care delivery implementation approaches are needed to improve cancer survivors‘ morbidity and mortality from both CV disease and cancer.

### Limitations

Although many cancer types were eligible for our study, breast cancer cases predominated, which may reflect the specialty of enrolling providers and the community medical oncology context, as well as the established role of breast cancer survivorship programs in community practices. Nevertheless, future research should seek to extend these findings to survivors of other prevalent cancer types (e.g., colorectal, lung, prostate). Due to the pragmatic nature of our trial, we also did not have data available about specific doses of chemotherapy agents and/or precise targets for radiation therapy, additional factors which may impact CVH.

### Electronic supplementary material

Below is the link to the electronic supplementary material.

Supplementary Material 1

Supplementary Material 2

## Data Availability

The datasets used and/or analysed during the current study are available from the corresponding author on reasonable request.

## Abbreviations

AH-HA: Automated Heart-Health Assessment
BP: Blood pressure
cIRB: NCI Central Institutional Review Board
CV: Cardiovascular
CVH: Cardiovascular health
EHR: Electronic health record
HbA1c: Hemoglobin A1c
NCCN Guidelines: NCCN Clinical Practice Guidelines in Oncology
NCORP: NCI Community Oncology Research Program
WF NCORP: Wake Forest NCI Community Oncology Research Program
