# Supplementary material for: Cardiovascular health assessment in routine cancer follow-up in community settings: survivor risk awareness and perspectives
Source: BMC Cancer. 2024 Jan 31;24:158. doi: 10.1186/s12885-024-11912-8 (PMC10829276; doi:10.1186/s12885-024-11912-8)
Supplement: Supplementary file 1 — Supplementary Material 1 [file 12885_2024_11912_MOESM1_ESM.docx]

Supplementary Material 2. Receipt of Cancer Treatments with Cardiac or Arrhythmia Toxicity Potential among Post-treatment Survivors

|  | **Any** | | **Treatments with Cardiotoxicity Frequency ≥1%** | | | **Treatments with Cardiotoxicity Frequency >10%** | | |  |
| --- | --- | --- | --- | --- | --- | --- | --- | --- | --- |
|  | **N** | **%** | | **N** | **%** | | **N** | **%** | |
| **Cancer Treatments** |  |  |  |  |  |  |  |  |  |
| Any | 471 | 93.8 | | 400 | 79.7 | | 153 | 30.5 | |
| Radiation | 341 | 67.9 | | 341 | 67.9 | |  |  | |
| Anthracyclines (i.e., Doxorubicin,  Valrubicin, Daunorubicin) | 116 | 23.1 | | 109 | 21.7 | | 109 | 21.7 | |
| Monoclonal antibodies (i.e.,     Trastuzumab, Rituximab) | 63 | 12.6 | | 53 | 10.6 | | 53 | 10.6 | |
| Antimicrotubule agents (i.e.,    Taxanes, Docetaxol ) | 216 | 43.0 | | 139 | 27.7 | | 0 | 0.0 | |
| Alkylating agents (i.e., Cisplatin,   Cyclophosphamide) | 224 | 44.6 | | 152 | 30.3 | | 0 | 0.0 | |
| Antimetabolites (5-fluorouracil,   Capecitabine) | 35 | 7.0 | | 7 | 1.4 | | 0 | 0.0 | |
| Hormone therapy (i.e., Tamoxifen,   Flutamide, Abiraterone,   Biclutamide, Enzalutamide) | 125 | 24.9 | | N/A | N/A | | N/A | N/A | |
| Aromatase inhibitors (i.e.,     Anastrozole, Letrozole, Exemestane) | 255 | 50.8 | | N/A | N/A | | N/A | N/A | |
| Other | 1 | 0.2 | | N/A | N/A | | N/A | N/A | |

Cardiotoxicity potential classified according to Herrmann 2020^36^
