# Supplementary material for: Cardiovascular health assessment in routine cancer follow-up in community settings: survivor risk awareness and perspectives
Source: BMC Cancer. 2024 Jan 31;24:158. doi: 10.1186/s12885-024-11912-8 (PMC10829276; doi:10.1186/s12885-024-11912-8)

Supplementary Material 2. Questions assessing the AHA Life’s Simple 7 Cardiovascular Risk Factors


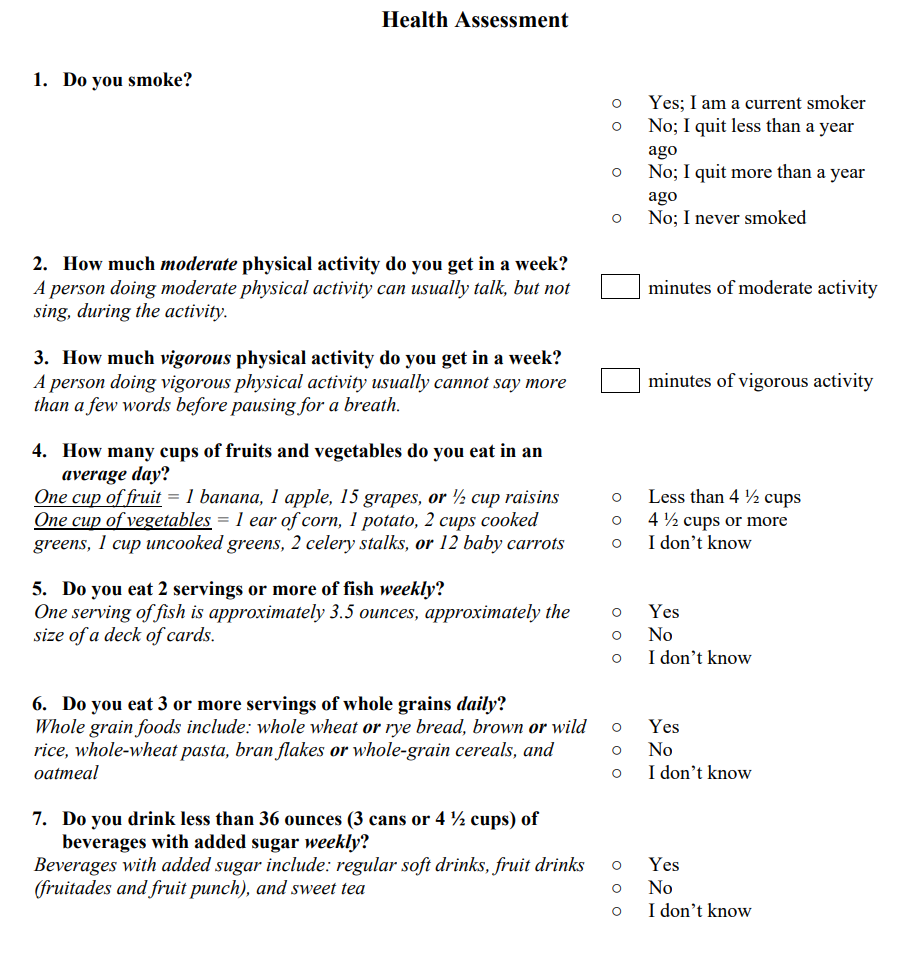


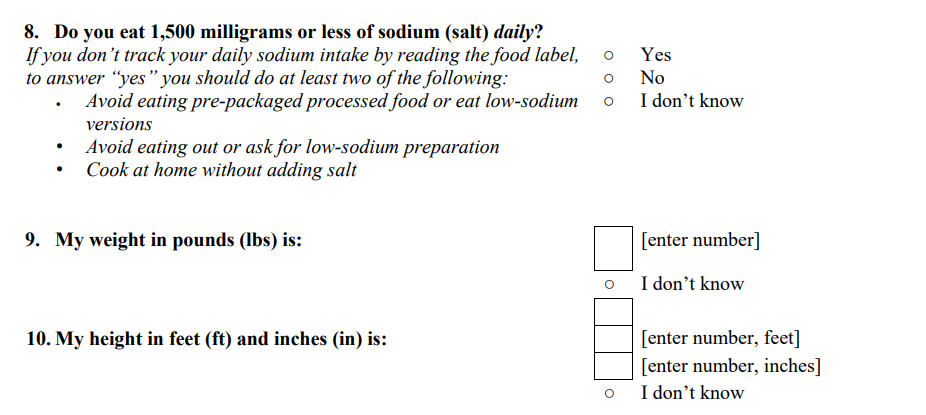


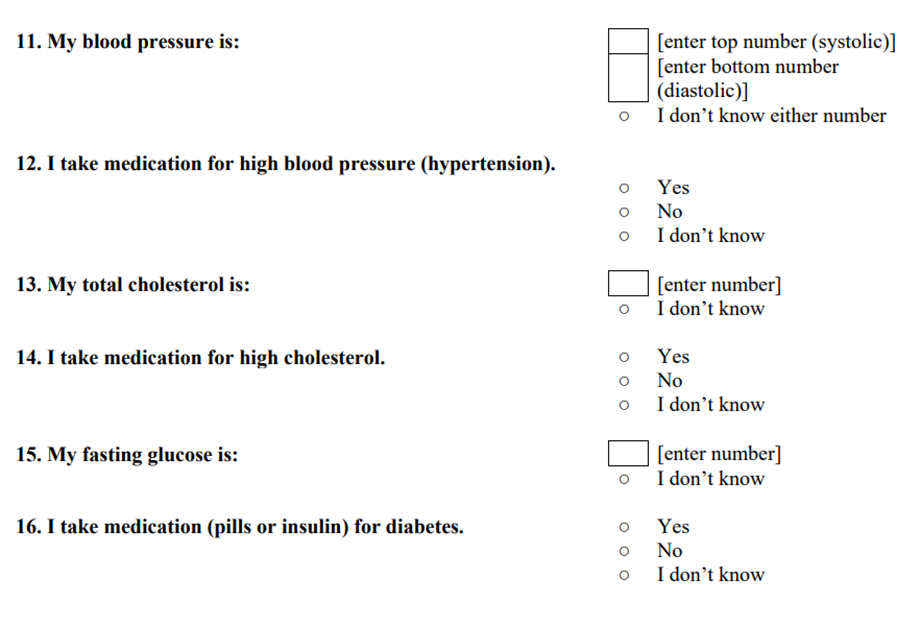

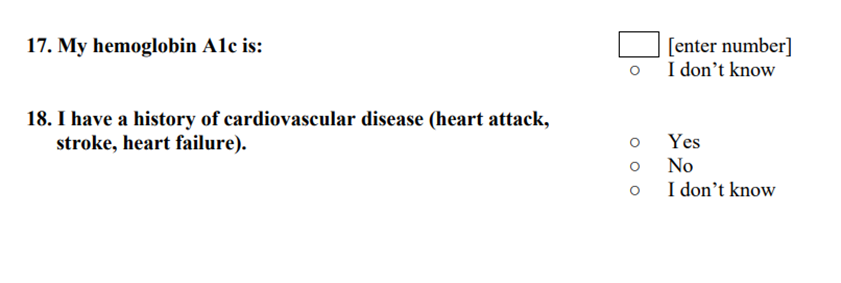

Supplement: Supplementary file 2 — Supplementary Material 2 [file 12885_2024_11912_MOESM2_ESM.docx]
